# Noninvasive evaluation of renal oxygenation by blood oxygenation level-dependent magnetic resonance imaging in patients with primary aldosteronism

**DOI:** 10.1038/s41598-025-18663-x

**Published:** 2025-09-26

**Authors:** Deying Wen, Ailin Liang, Xun Yue, Pengfei Peng, Chenxiao Xu, Qian Pu, Yue Ming, Yue Qiu, Miaoqi Zhang, Bo Zhang, Lin He, Yan Ren, Jiayu Sun

**Affiliations:** 1 Department of Radiology, West China Hospital, Sichuan University, Chengdu, China; 2 Department of Radiology, Affiliated Hospital of North Sichuan Medical College, Nanchong, China; 3 Department of Endocrinology and Metabolism, West China Hospital, Sichuan University, Chengdu, China; 4 GE Healthcare, MR Research, Beijing, China; 5 Library and Information Center, West China Hospital, Sichuan University, Chengdu, China

**Keywords:** Primary aldosteronism, Blood oxygenation level-dependent magnetic resonance imaging, Kidney oxygenation, Endocrinology, Adrenal gland diseases, Magnetic resonance imaging

## Abstract

This study investigated renal oxygenation status in primary aldosteronism (PA) patients using blood oxygen level-dependent magnetic resonance imaging (BOLD-MRI), with comparative analysis against healthy controls and correlation assessments with biochemical markers of renal function. A total of 48 patients and 27 healthy controls were enrolled. All participants underwent renal BOLD-MRI with a 3 T MRI scanner. The R2* values were measured in the renal cortex and medulla of the bilateral kidneys using the region of interest method. Paired-sample t tests and independent-samples t tests were used. Pearson correlation analysis examined the relationship between R2* values and clinical indicators, and ROC curve analysis evaluated the performance of R2* values in distinguishing PA patients from healthy controls. The cortical R2* values were significantly lower than the medullary R2* values for all participants. For PA patients, left kidney cortical and medullary R2* values were significantly higher than those of the right kidney. Left kidney cortical R2* values in PA patients were significantly higher than in healthy controls. Medullary R2* values positively correlated with blood urea nitrogen (*r* = 0.408, *p* = 0.005). The optimal threshold for left cortical R2* in discriminating PA patients from healthy controls was 18.955 Hz, yielding a sensitivity of 75.5% and a specificity of 63.0%, with an AUC of 0.717 (95% CI, 0.593–0.841). BOLD-MRI can detect renal hypoxia in patients with PA, suggesting its potential as a noninvasive tool for renal assessment. Specifically, the cortical R2* value in the left kidney demonstrated a moderate ability (AUC = 0.717) to distinguish PA patients from healthy controls.

## Introduction

Primary aldosteronism (PA) is a common endocrine disorder characterized by excessive aldosterone production from the adrenal cortex, leading to clinical manifestations such as persistent hypertension and hypokalemia. Renal injury, a significant complication for PA patients, is intimately associated with prolonged hypertension and aldosterone overproduction. The physiological impact of aldosterone, which involves increasing renal sodium reabsorption and subsequent increases in blood volume and hypertension, places an added burden on the kidneys. These processes can further result in abnormal vasodilation, renal vascular damage, microvascular impairments, and endothelial dysfunction, culminating in inadequate renal oxygenation and hypoxic damage. Over time, this persistent hypoxic state can cause irreversible renal damage, which may progress to chronic renal insufficiency^1–3^. Prior studies have indicated that PA patients face a greater risk of renal impairment and of developing chronic renal insufficiency than those with essential hypertension^4^.

In clinical practice, the estimated glomerular filtration rate (eGFR) is a widely accepted metric for renal function. However, in patients with PA, the excess secretion of aldosterone stimulates sodium reabsorption and subsequent extracellular volume expansion, leading to increased renal perfusion pressure and hyperfiltration of the glomeruli^3^. This results in an overestimation of the eGFR, making it an inaccurate reflection of the true renal function in PA patients. Therefore, it is possible that even when the eGFR values are within the normal range, renal injury may already be present. Additionally, eGFR is based on serum creatinine levels, which can be influenced by various factors such as muscle mass, diet, and certain medications. Moreover, eGFR provides an overall estimate of renal function and cannot assess the function of individual kidneys. Consequently, an early noninvasive assessment of renal oxygenation in PA patients is important for evaluating renal injury, enabling timely clinical intervention, and halting the progression of renal damage.

Blood oxygen level-dependent magnetic resonance imaging (BOLD-MRI) is a specialized functional MRI technique that quantifies tissue oxygenation by exploiting magnetic susceptibility effects. The technique leverages the paramagnetic properties of deoxyhemoglobin: deoxygenated hemoglobin generates local magnetic field inhomogeneities, which accelerate proton spin dephasing. This shortens the transverse relaxation time (T2*), thereby increasing the apparent relaxation rate R2* (R2* = 1/T2*). Elevated R2* values directly reflect an increased concentration of deoxygenated hemoglobin, indicating reduced tissue oxygenation. BOLD-MRI has previously been demonstrated to serve as a reliable method for measuring renal oxygenation^5^. To our knowledge, it is the only MRI-based technique for the noninvasive assessment of renal oxygenation status and has been applied to a wide range of renal diseases, such as diabetic nephropathy^6,7^, chronic kidney disease^8,9^, kidney transplantation^10^, and lupus nephritis^11^. However, studies employing BOLD-MRI in PA patients are relatively rare, and the relationship between renal oxygenation status and the pathophysiology of PA therefore remains insufficiently elucidated.

This study aimed to evaluate renal oxygenation status in PA patients using BOLD-MRI, exploring its association with renal functional impairment, with the goal of providing new perspectives and scientific evidence for the assessment and treatment of PA patients. Assessing renal oxygenation status using BOLD-MRI helps clinicians promptly identify potential kidney damage, allowing for timely adjustments to treatment plans and providing a basis for personalized treatment.

## Materials and methods

### Study population

The prospective study was conducted in accordance with the Declaration of Helsinki (as revised in 2013), and was approved by the Biomedical Ethics Committee of West China Hospital, Sichuan University (No. 2019 145). All the participating patients provided written informed consent before undergoing the examination.

A total of 54 patients with a confirmed diagnosis of PA were included from June 2021 to December 2023. The patient inclusion criteria were as follows: (1) a diagnosis of primary aldosteronism in accordance with the 2016 American Endocrine Society Clinical Practice Guidelines^12^, which required the presence of following: (1). Spontaneous hypokalemia, a plasma renin concentration below detection limits, and a plasma aldosterone concentration (PAC) exceeding 20 ng/dL; and (2). A positive plasma aldosterone-to-renin ratio (ARR) accompanied by one or more affirmative confirmatory tests, including the captopril challenge test and saline infusion test; (2) No specific therapeutic interventions (such as adrenalectomy or mineralocorticoid receptor antagonists) post-PA diagnosis; (3) An age of 18 years or older; and (4) The absence of other renal pathologies or conditions impacting renal function.

A total of 28 healthy control individuals were enrolled. The inclusion criteria for the healthy volunteers were as follows: (1) No intake of medications that could influence the physiological functioning of the kidneys and blood vessels within the past three months; (2) No history of surgical procedures, systemic infections, or severe traumatic events in the preceding six months; and (3) The absence of chronic diseases, including neurological, cardiovascular, respiratory, and immune system disorders.

The exclusion criteria for all participants were as follows: (1) Poor-quality magnetic resonance (MR) images that precluded analysis; (2) Contraindications to MR imaging, such as claustrophobia or other psychiatric conditions, that could interfere with compliance during MR examinations; (3) Presentation with concomitant renal diseases or discernible lesions on the kidneys; and (4) For PA patients, an inability to temporarily discontinue or switch medications.

**Fig. 1:**
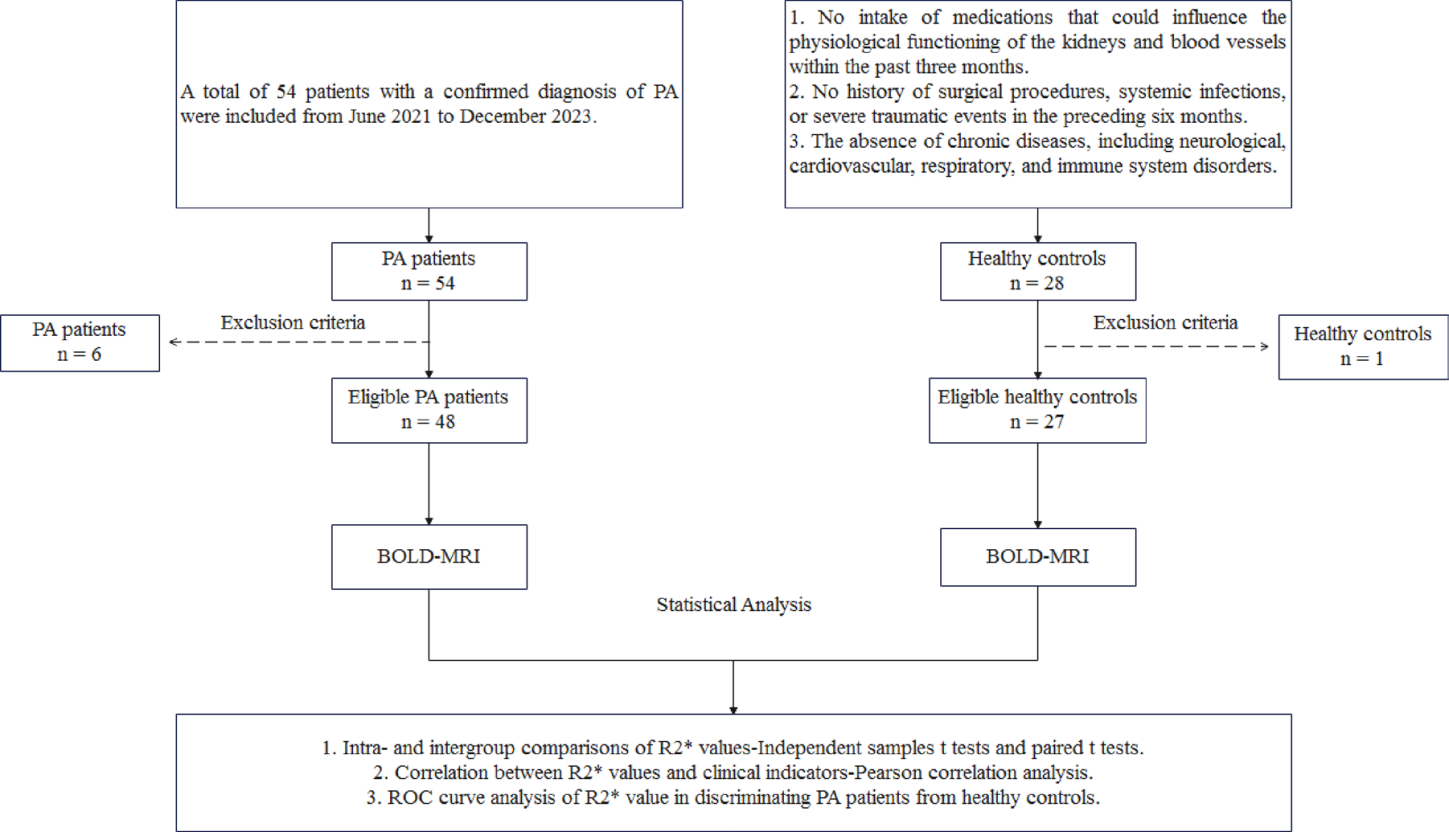
Study flow diagram. PA, primary aldosteronism; ROC, receiver operating characteristic; BOLD-MRI, blood oxygenation level-dependent magnetic resonance imaging.

Finally, a total of 48 PA patients and 27 healthy volunteers who met the inclusion and exclusion criteria were included. The study flow diagram is presented in Fig. 1.

### Clinical data

Biochemical parameters including PAC, direct renin concentration (DRC), ARR, potassium, blood urea nitrogen (BUN), creatinine, uric acid, serum cystatin C and eGFR, were collected and recorded for all PA patients the day before the MR examination. Basic information for all participants, including sex, age, height, weight, body mass index (BMI), systolic blood pressure (SBP) and diastolic blood pressure (DBP), was recorded.

### Preparation for BOLD-MRI examination

Prior to the MR examination, it was essential to first measure clinical biochemical indicators. According to the 2016 American Endocrine Society Clinical Practice Guidelines^12^, it was necessary for the participants to discontinue medications that significantly affect the ARR for at least four weeks, including potassium-sparing and potassium-wasting diuretics. Medications that influence renin activity, including angiotensin-converting enzyme inhibitors (ACEIs), angiotensin receptor blockers (ARBs), and calcium channel blockers (CCBs), had to be stopped at least two weeks before the test. Furthermore, any medication that may impact renal function had to be discontinued or substituted with equivalent treatments that would not affect renal function. All temporary cessation or changes in medication were determined by a professional clinician based on the patient’s specific condition. During this period, trained nurses or doctors regularly contacted the patients by phone and instructed them to contact us immediately if they experienced any adverse symptoms. For certain patients admitted to our institution, daily routine ward rounds were capable of promptly detecting any abnormalities. After the testing period, patients were allowed to resume their original medication under the supervision of a professional clinician. If a professional clinician determined that the patient could not discontinue or switch medications, the patient was instructed to maintain their current treatment and was excluded from the study.

### MRI acquisition

All participants underwent BOLD-MRI examination within one week of PA diagnosis. Before the MR examination, all participants were instructed to fast and refrain from consuming water for 4 to 6 h. The scans were conducted with the participants in a supine position utilizing a 3.0 Tesla MRI scanner (Signa Architect, GE Healthcare). The scan sequences employed were as follows: axial T1-weighted imaging, axial and coronal T2-weighted imaging and coronal BOLD imaging. Participants were instructed to hold their breath during each scan sequence, with each scan lasting approximately 15 to 20 s. BOLD MR imaging was performed by using a multiple gradient-recalled-echo sequence with fat suppression. Eight slices were acquired per breath-hold across two consecutive breath-holds, yielding 16 slices in total. The acquisition parameters for the BOLD imaging sequence were as follows: number of echoes, 8; echo time (TE), 1.20, 2.33, 3.46, 4.58, 5.71, 6.84, 7.97, 9.10 ms; repetition time (TR), 36.60 ms; flip angle, 25°; number of slices, 16; slice thickness, 4 mm; interval, 0.5 mm; field of view (FOV), 30 × 30 cm^2^; matrix, 96 × 96; bandwidth, 1736 Hz/Pixel; number of excitations (NEX), 1; parallel acceleration factor, 1; and partial Fourier factor, 1.

### Image analysis

**Fig. 2:**
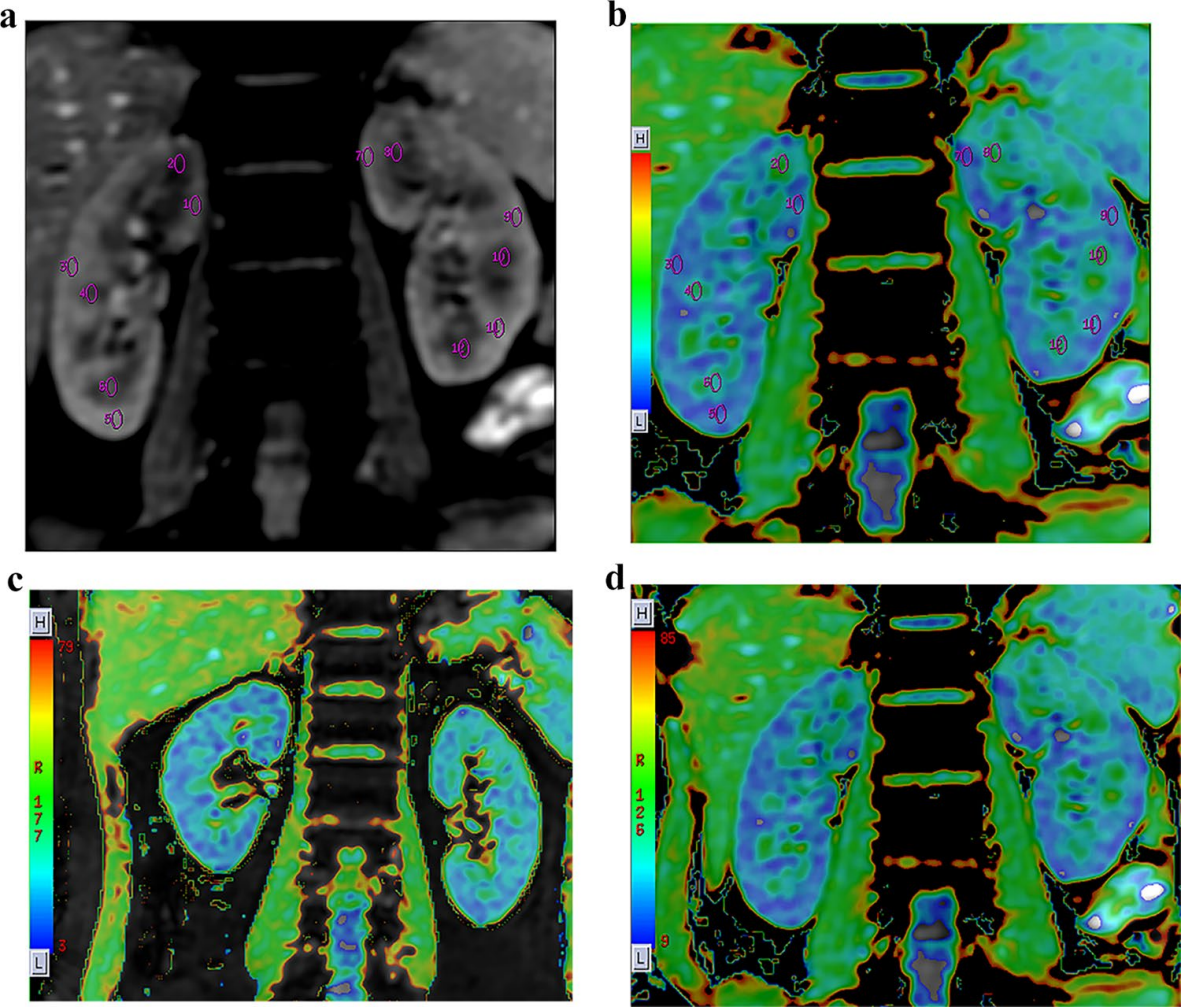
Schematic representation of ROI placement and representative R2* maps. (**A**) Grayscale image demonstrating precise ROI positioning within the renal cortex (R:1,3,5; L: 7,9,11) and medulla (R: 2,4,6; L: 8,10,12). (**B**) Corresponding parametric R2* map with identical ROIs superimposed (validating parenchymal localization). (**C**) R2* map of a representative PA patient showing elevated cortical R2* (left > right). (**D**) R2* map of a healthy volunteer demonstrating physiological corticomedullary R2* gradient. Color scale: R2* values in s^−^¹ (blue: low oxygenation; red: high oxygenation). All images acquired at 3T with identical parameters. ROI, regions of interest; PA, primary aldosteronism.

After completion of the scanning sequences, the raw imaging data were transferred to a workstation (Advanced Workstation 4.6), where the R2* values were calculated using BOLD software within the Functool toolkit. Two independent observers blinded to participants’ clinical data independently delineated elliptical regions of interest (ROIs) on grayscale images using a standardized protocol^13^ as follows: (1) A coronal image at the central level of the renal hilum in which the boundary between the renal cortex and medulla could be clearly discerned was selected for analysis. (2) Oval-shaped ROIs were positioned in the upper, middle, and lower sections of both the cortex and medulla for each kidney. (3) The diameters of the ROIs were kept within the boundaries of the thickness of the renal cortex as displayed on the original image, with each ROI covering an approximate area of 20–40 mm^2^. (4) Special care was taken to avoid the peripheral edges of the kidneys, the junction between the cortex and medulla, the renal sinus, and any large blood vessels. (5) The R2* values for the renal cortex and medulla were reported as the mean of the measurements taken from the ROIs delineated within the upper, middle, and lower sections. Schematic illustrations of the ROIs are presented in Fig. 2A-B.

### Statistical analysis

The distribution of the variables was assessed using the Shapiro-Wilk test. Normally distributed data are presented as mean ± standard deviation, and non-normally distributed data as median and interquartile range. The intraclass correlation coefficient (ICC) was used to assess inter-observer consistency for measurements. Independent samples t-tests were used to compare variables between the cortex and medulla, and between PA patients and healthy controls (HCs). Paired t-tests were used to compare R2* values between the right and left kidneys. Cohen’s d was computed for all t-tests to quantify effect sizes, with conventional interpretive thresholds applied: d ≈ 0.2 (small effect), d ≈ 0.5 (medium effect), and d ≈ 0.8 (large effect). Pearson correlation analysis was used to assess the relationship between renal BOLD parameters and clinical indicators. ROC curve analysis was performed to evaluate the diagnostic performance of R2* values in differentiating PA patients from HCs, with AUC quantifying the performance. The optimal cutoff point for maximizing sensitivity and specificity was determined using the Youden index. Statistical analysis was performed with SPSS 25.0 (IBM, Corp.) and MedCalc (version 20.218) statistical software. *P* < 0.05 indicated statistical significance for all tests.

## Results

### Clinical characteristics

A total of 54 patients with a confirmed diagnosis of PA were enrolled in this prospective study between June 2021 and December 2023. Six patients were excluded due to poor image quality from respiratory motion artifacts (*n* = 2), concomitant renal cysts (*n* = 2), or mild claustrophobia (*n* = 1), leaving 48 eligible cases (29 females and 19 males; mean age 50.10 ± 11.63 years). Following exclusion of one healthy volunteer with significant respiratory motion artifacts, 27 healthy volunteers (16 females and 11 males; mean age 46.22 ± 6.72 years) were ultimately included as controls. There were significant differences in BMI, SBP, and DBP between the PA patients and HCs. The clinical characteristics of the study participants are summarized in Table 1.

**Table 1:** Demographic and clinical characteristics of PA patients and healthy controls.

	PA	HCs	*p*
Sex (male: female)	19:29	11:16	
Age, years	50.10 ± 11.63	46.22 ± 6.72	0.107
BMI, kg/m^2^	25.21 ± 3.48	22.36 ± 2.63	0.001
SBP, mmHg	151 ± 19	111 ± 4	< 0.001
DBP, mmHg	98 ± 12	76 ± 5	< 0.001
PAC, ng/dL	27.30 (18.40–49.30)		
DRC, µIU/mL	2.76 (1.01–5.78)		
ARR, ng/dL:µIU/mL	8.00 (3.90-27.47)		
Potassium, mmoL/L	3.24 ± 0.58		
BUN, mmoL/L	4.47 ± 1.21		
Creatinine, µmoL/L	68.76 ± 14.69		
Uric acid, µmoL/L	331.48 ± 80.55		
Serum cystatin C, mg/L	0.97 ± 0.16		
eGFR, mL/min/1.73m^2^	97.21 ± 15.41		

**Notes**: Data are presented as the mean ± standard deviation and median (interquartile range), and sex is presented as the number of individuals. BMI, body mass index; SBP, systolic blood pressure; DBP, diastolic blood pressure; PAC, plasma aldosterone concentration; DRC, direct renin concentration; ARR, plasma aldosterone-to-renin ratio; BUN, blood urea nitrogen; eGFR, estimated glomerular filtration rate; PA, primary aldosteronism; HCs, healthy controls.

### Inter-rater reliability of R2* measurements

Inter-rater reliability for renal R2* measurements was quantified using intraclass correlation coefficients, demonstrating good agreement (ICC = 0.837, 95% CI: 0.715–0.906).

### Intra- and intergroup comparisons of R2* values

Across all subjects, the R2* values in the renal cortex were significantly lower than those in the medulla (*p* < 0.01). For PA patients, the R2* values were greater in the left kidney for both the cortex and medulla than in the right kidney. However, among the HCs, the R2* values did not differ significantly between the two kidneys. Furthermore, the R2* values in the cortex of the left kidney (L-Cortical R2*) of PA patients were substantially greater than those in the HC group. These results are presented in detail in Tables 2 and 3, and representative R2* maps for PA patients and healthy controls are depicted in Fig. 2.

**Table 2:** Intragroup comparisons of R2* values.

		Cortical R2*	Medullary R2*	*p* ^#^	Cohen’s d^#^
PA	R	19.814 ± 3.032	27.047 ± 3.568	< 0.001	2.18
	L	21.238 ± 3.303	28.359 ± 4.093	< 0.001	1.91
	*p* ^*^*^	0.02	0.027		
	Cohen’s d^*^*^	0.35	0.33		
HCs	R	18.744 ± 4.466	28.602 ± 3.968	< 0.001	2.33
	L	18.406 ± 3.321	28.930 ± 5.766	< 0.001	2.24
	*p* ^*^*^	0.720	0.778		
	Cohen’s d^*^*^	0.07	0.06		

**Notes**: Data are presented as the mean ± standard deviation. The unit of R2* is Hz.

^#^, comparison of R2* values between cortex and medulla; ^^^, comparison of R2* values between the right and left kidneys. PA, primary aldosteronism; HCs, healthy controls.

**Table 3:** Comparison of R2* values between PA patients and healthy controls.

	*R*-Cortical R2*	*R*-Medullary R2*	L-Cortical R2*	L-Medullary R2*
PA	19.814 ± 3.032	27.047 ± 3.568	21.238 ± 3.303	28.359 ± 4.093
HCs	18.744 ± 4.466	28.602 ± 3.968	18.406 ± 3.321	28.930 ± 5.766
*p*	0.271	0.082	0.001	0.617
Cohen’s d	0.28	0.41	0.86	0.11

**Notes**: Data are presented as the mean ± standard deviation. The unit of R2* is Hz.

PA, primary aldosteronism; HCs, healthy controls; R-Cortical R2*, cortical R2* value in the right kidney; R-Medullary R2*, medullary R2* value in the right kidney; L-Cortical R2*, cortical R2* value in the left kidney; L-Medullary R2*, medullary R2* value in the left kidney.

### Correlation between R2* values and clinical indicators

**Fig. 3:**
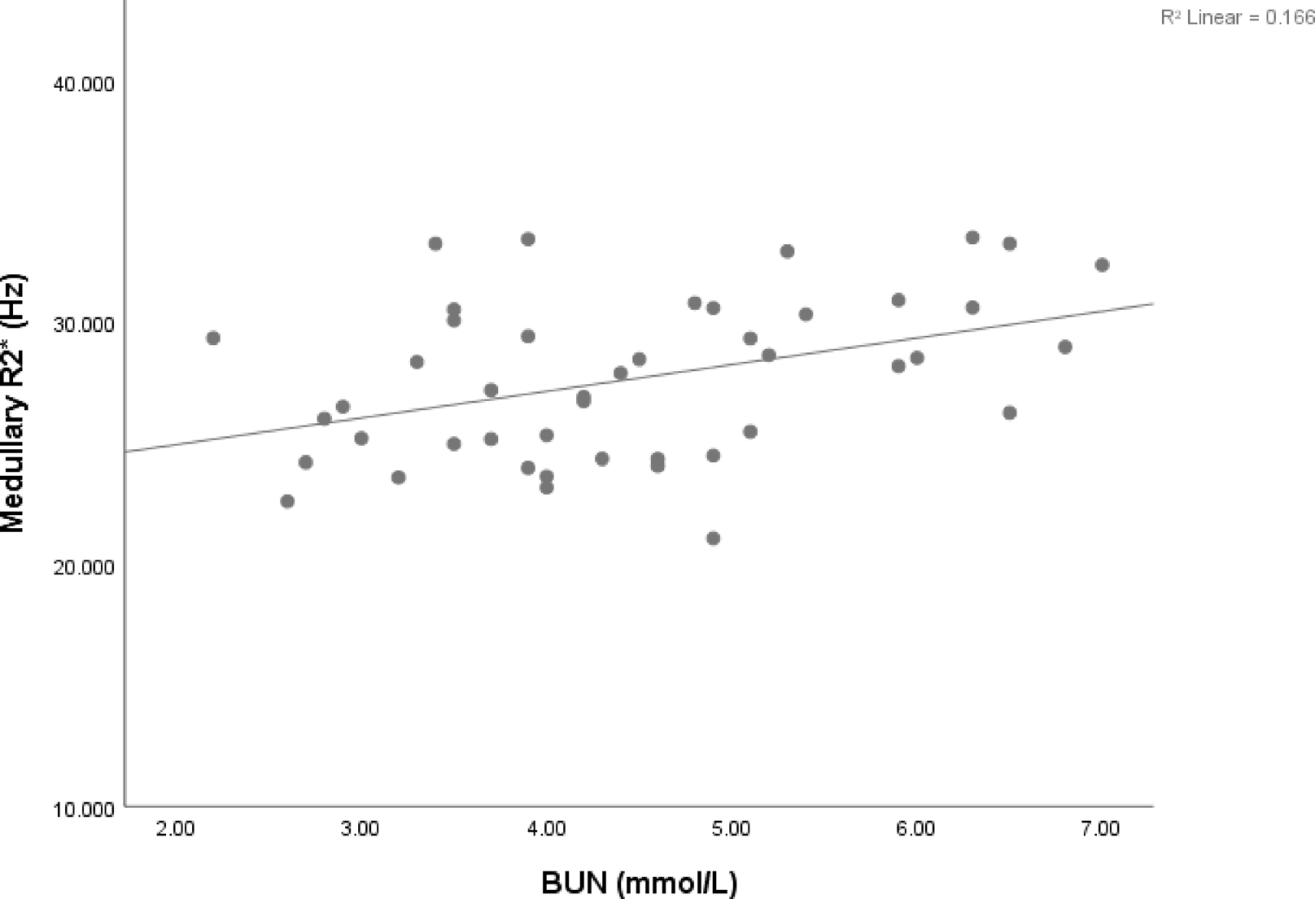
Correlation analysis between R2* values and clinical characteristics. Correlation analysis revealed a significant and positive correlation between the medullary R2* value and BUN level (*r* = 0.408, *p* = 0.005). BUN, blood urea nitrogen.

We conducted correlation analyses between the R2* values in the cortex and medulla and biochemical indicators reflecting renal function, including BUN, creatinine, uric acid, serum cystatin C, and eGFR. Our analyses revealed a significant and positive correlation between the medullary R2* value and BUN (*r* = 0.408, *p* = 0.005). The results of the correlation analysis are displayed in Fig. 3.

### ROC curve analysis of L-Cortical R2* value in discriminating PA patients from healthy controls

**Fig. 4:**
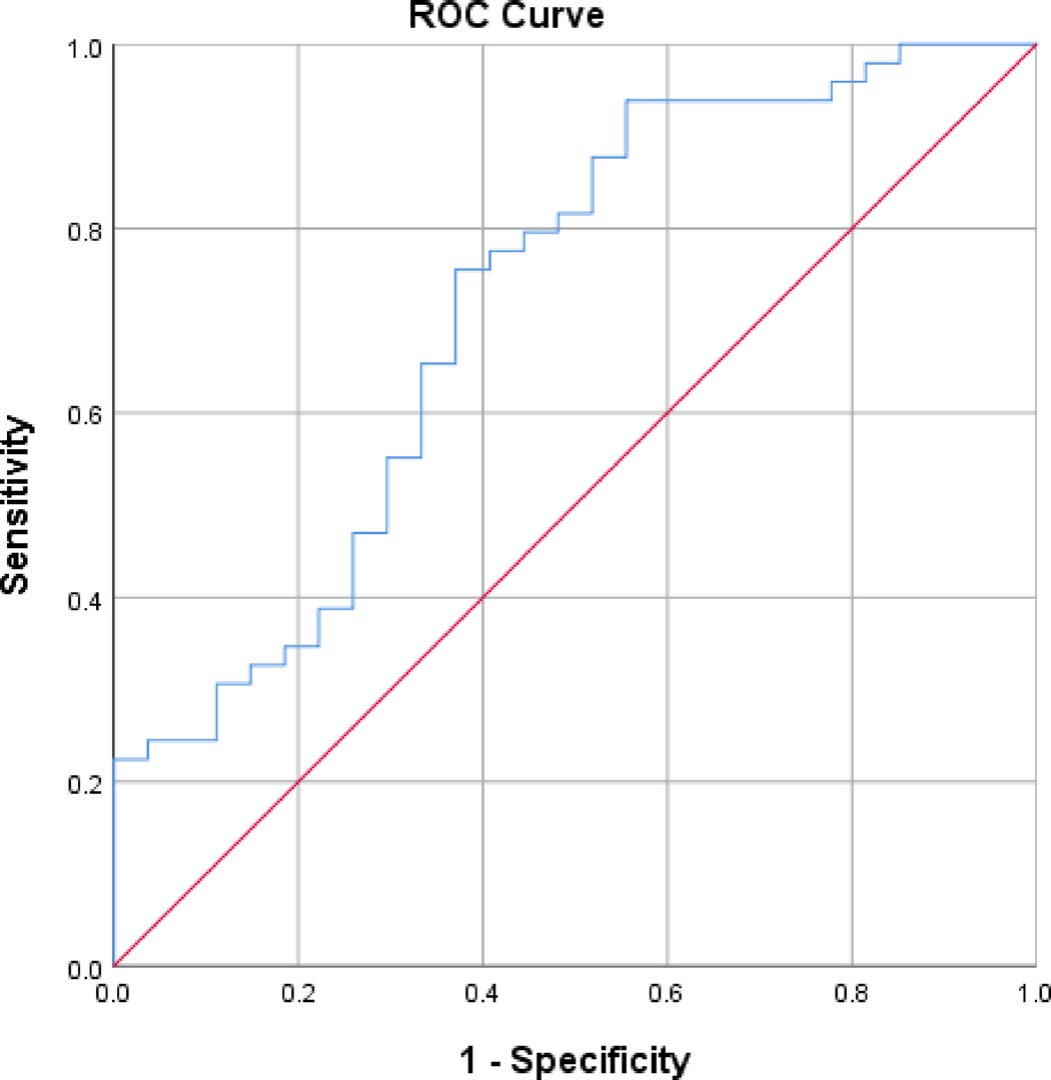
ROC curve analysis for L-Cortical R2* in discriminating PA patients from healthy controls. ROC curve analysis indicated that the optimal cutoff value of L-Cortical R2* in differentiating PA patients from healthy controls was 18.955 Hz, yielding a sensitivity of 75.5% and a specificity of 63.0%, with an AUC of 0.717 (95% CI, 0.593–0.841). ROC, receiver operating characteristic; PA, primary aldosteronism; AUC, area under the curve.

In comparing the healthy control group and PA patients, significant differences were observed in the L-Cortical R2* value. Consequently, we subjected this value to ROC curve analysis to assess its diagnostic efficacy in distinguishing PA patients from HCs. The results of the ROC curve analysis indicated that the optimal cutoff value for L-Cortical R2* in differentiating PA patients from HCs was 18.955 Hz, yielding a sensitivity of 75.5% and a specificity of 63.0%, with an AUC of 0.717 (95% CI, 0.593–0.841).These results are presented in Fig. 4.

## Discussion

This study employed BOLD-MRI to noninvasively assess renal oxygenation in patients with PA compared to healthy controls. Our key findings demonstrated that: (1) PA patients exhibited significantly higher cortical R2* values in the left kidney compared to HCs, indicative of cortical hypoxia specifically in the left kidney; (2) within PA patients, both cortical and medullary R2* values were significantly higher in the left kidney than in the right kidney; (3) medullary R2* values showed a significant positive correlation with BUN levels; and (4) L-Cortical R2* demonstrated potential as a discriminatory biomarker between PA patients and HCs, achieving an AUC of 0.717. These results collectively suggest that BOLD-MRI effectively detects alterations in renal oxygenation associated with PA, particularly highlighting a state of relative hypoxia in the left renal cortex.

In this study, we observed that the R2* values of the left kidney in PA patients were significantly greater than those of the right kidney. This finding aligns with the established physiological asymmetry reported in healthy populations^14,15^, where anatomical factors (e.g., compression of the left renal vein between the aorta and superior mesenteric artery, shorter right renal vein length) typically result in reduced left renal perfusion and higher R2* values. While this inherent anatomical asymmetry exists universally, its statistical detectability in healthy cohorts can be influenced by sample size and methodological factors. In our study, the lack of statistically significant left-right R2* difference in the HC group (*n* = 27) may reflect limited power to resolve this subtle physiological variation. The amplification observed in PA patients likely results from disease-specific mechanisms (aldosterone-induced reductions in cortical perfusion^16^ and increased oxygen consumption^17–20^ impacting the pre-existing anatomically disadvantaged left kidney. This exacerbation pushes the left kidney’s hypoxia beyond the detection threshold more readily than the right, making the asymmetry statistically apparent in the larger PA cohort (*n* = 48). Importantly, the preservation of the fundamental left > right R2* relationship in PA patients (Table 2) indicates that aldosteronism impacts renal oxygenation synchronously across both kidneys, rather than selectively targeting one side. The disease appears to worsen hypoxia globally, but its effects are more readily measurable in the already vulnerable left cortex. This interpretation is supported by the trend of higher R2* values in the right cortex of PA patients compared to HCs (Table 3), although this difference did not reach statistical significance, potentially due to the right kidney’s more favorable baseline oxygenation state or the cohort’s disease stage. Whether PA induces characteristic changes beyond simply exacerbating the anatomical predisposition requires further investigation. Notably, some studies found no asymmetry in chronic kidney disease^21^ or diabetic nephropathy^6^, potentially due to small samples, advanced disease stages homogenizing oxygenation, or distinct pathophysiologies altering vascular dynamics.

The R2* values observed in the renal medulla were significantly greater than those in the cortex across all study participants, consistent with the findings of several earlier studies^8,9,22^. This pattern suggests that the oxygen content in the renal cortex is substantially greater than that in the medulla, an observation that can be inferred from the intrinsic physiological structure of the kidneys and the disparity in blood flow distribution between the cortex and medulla. Under typical physiological conditions, the renal blood flow is approximately 1200 ml/minute, the equivalent to 20–25% of the cardiac output. Approximately 94% of this total is directed toward the renal cortex, leaving a mere 6% for the renal medulla. Consequently, this distribution pattern predisposes the renal medulla to a more pronounced physiological hypoxic state than does the cortex. Under healthy conditions, the partial pressure of oxygen is approximately 50 mmHg in the cortex and between 10 and 20 mmHg in the medulla^23^. Additionally, the active reabsorption mechanisms in the renal tubules contribute to relatively high oxygen consumption in the medulla, further exacerbating its physiological hypoxic state^24^.

In our study, we found that the L-Cortical R2* values were significantly greater in the PA patient group than in the healthy control group, indicating a state of relative hypoxia in the renal cortices of the left kidney in PA patients. Individuals with PA are characterized by the autonomous overproduction of aldosterone from the adrenal cortex. Increased levels of aldosterone exert direct nephrotoxic effects^18–20^, including (1) damage to podocytes, which leads to glomerulosclerosis; (2) an impact on vascular smooth muscle cells, resulting in vascular hypertrophy and hyalinization of small arteries; and (3) endothelial cell swelling and dysfunction, contributing to increased vascular stiffness or peripheral resistance and, consequently, diminished renal blood flow. These factors directly reduce the renal blood supply to the cortex and lead to a state of relative hypoxia due to a decrease in oxygen delivery. Additionally, several studies have confirmed the presence of a state of glomerular hyperfiltration state in PA patients^25–28^, which results in an increase in kidney oxygen consumption. This combination of compromised oxygen transport and elevated oxygen demand contributes to hypoxia in the renal cortex and an increase in its R2* value^17^. As discussed previously, the anatomically predisposed left kidney exhibits greater susceptibility to this hypoxia. Consequently, R2* differences between PA patients and controls were statistically detectable only in the left cortex, consistent with its heightened vulnerability. While this lateralization aligns with physiological patterns, its precise mechanisms warrant further study. Notably, there was no significant difference in the medullary R2* values between patients with PA and healthy volunteers. We propose several factors that may account for this observations: (1). The sample size was insufficient for detecting subtle changes in the medulla with statistical analysis; (2). The renal cortex is primarily responsible for filtration and absorption, possessing a rich vascular distribution to meet high metabolic demands, whereas the medulla is more involved in urine concentration and contains a sparser vascular distribution; the reduction in blood supply due to various pathological causes in PA patients has a less noticeable impact on the medullary region than on the cortical region, leading to the inability to detect changes in the former; (3). This aligns with our previous finding from a multi-parametric diffusion study comparing PA patients and healthy volunteers^16^, where we observed elevated α-values in PA patients, potentially attributable to reduced cortical perfusion, while medullary a-values showed no significant difference. Consequently, we conducted ROC curve analysis for L-Cortical R2* to assess its ability to discriminate between patients and healthy individuals. This analysis revealed that the optimal cutoff value for L-Cortical R2* in differentiating the two groups was 18.955 Hz; the AUC, specificity and sensitivity were 0.717, 63.0% and 75.5%, respectively. However, this threshold is context-specific, derived under controlled experimental conditions. Its clinical translation requires addressing technical variability (e.g., field strength and specific sequence parameters dependence ) and validating in diverse populations with comorbidities. Future studies correlating R2* thresholds with treatment response may strengthen its biological relevance.

In clinical practice, the eGFR is commonly measured for evaluating renal function; however, our study did not find a correlation between BOLD-MRI parameters and the eGFR, which could be attributed to the presence of glomerular hyperfiltration in patients with PA. This hyperfiltration phenomenon can cause an overestimation of eGFR, making it an inaccurate reflection of true renal function. Interestingly, we did observe a significant correlation between the medullary R2* value and BUN. Blood urea nitrogen is a byproduct of protein catabolism and metabolism, of which over 90% being excreted by the kidneys, while the remainder is excreted through the intestines and skin. The level of this product may increase when the excretory function of the kidneys is impaired by various pathologies and thus serves as a vital indicator of renal function alterations. The correlation between elevated medullary R2* and BUN levels may stem from aldosterone-induced tubular injury, which exacerbates medullary hypoxia and disrupts urea transporter function. As BUN is reabsorbed primarily in the medullary collecting ducts, hypoxia-driven impairment of active transport mechanisms reduces urea clearance, establishing a pathophysiological link between medullary oxygenation status and tubular functional reserve. This finding offers a novel perspective for clinically monitoring and evaluating renal function in PA patients based on their renal oxygenation status. However, no correlation was found between the cortical R2* value and the level of BUN, which could be attributed to the different oxygen sensitivities between the cortex and medulla; the medulla is more sensitive to minor changes in oxygen partial pressure because the oxygen level in medullary blood falls within the linear portion of the hemoglobin oxygenation curve, whereas the cortical oxygen level is situated on the plateau of the curve^8,29^.

Our study had several limitations. First, the sample size was relatively small, mainly due to our strict inclusion and exclusion criteria. Over the course of more than two years, from June 2021 to December 2023, we recruited only 48 patients with PA. Our findings therefore need to be validated through larger cohort studies. Second, we employed the traditional and commonly used manual ROI method; however, some studies have suggested that the semiautomated twelve-layer concentric objects (TLCO) method may be preferable due to reduced interobserver variability. Nevertheless, the regional R2* measurements obtained by both the ROI and TLCO methods are generally in good agreement^30^. Third, our dataset lacked information on the patients following their PA treatment, including adrenalectomy and mineralocorticoid receptor antagonist administration; thus, future follow-up studies are warranted. Fourth, the absence of a control group of patients with essential hypertension displaying blood pressure levels comparable to those of the PA patients prevented us from discerning whether renal injury was predominantly due to aldosterone excess or hypertension. Future studies should aim to include such a control group to better determine the specific cause of renal injury. Fifth, the requirement for antihypertensive medication withdrawal (e.g., ACEIs, ARBs, CCBs) may select for milder PA presentations, as patients with severe uncontrolled hypertension could not safely discontinue medications. This aligns with diagnostic standards minimizing pharmacological interference with ARR biomarkers^12^. Although our cohort included patients with significant hypertension severity (SBP range: 100–188 mmHg), future studies should explore inclusive strategies to mitigate this bias while maintaining diagnostic validity. Furthermore, While while healthy controls underwent comprehensive health screening, the absence of quantitative renal biomarkers (e.g., eGFR) represents a limitation. Future studies should include these measures to further validate control cohort homogeneity.

In summary, our study provides evidence that BOLD-MRI can detect alterations in renal oxygenation levels in patients with PA, suggesting its potential as a noninvasive tool for renal assessment. Specifically, the L-Cortical R2* value derived from BOLD-MRI demonstrated a moderate ability (AUC = 0.717) to distinguish PA patients from healthy controls.

## Data Availability

All data generated or analysed during this study are included in this published article. De-identified patient data will be shared with qualified academic researchers upon reasonable request to corresponding author (cardiac_wchscu@163.com), starting from the publication date for a period of two years. Data sharing is contingent on institutional review board approval, signed data access agreements, and non-commercial use. Study protocol and statistical analysis plan will be provided if requested. All shared data are subject to risk-benefit assessment per institutional policies.
